# A highly thermotolerant laccase produced by *Cerrena unicolor* strain CGMCC 5.1011 for complete and stable malachite green decolorization

**DOI:** 10.1186/s13568-020-01118-z

**Published:** 2020-10-02

**Authors:** Yanhua Yao, Guimei Zhou, Yonghui Lin, Xinqi Xu, Jie Yang

**Affiliations:** 1 Fujian Key Laboratory of Marine Enzyme Engineering, College of Biological Sciences and Technology, Fuzhou University, Fuzhou, 350116 Fujian China; 2 GRG Metrology & Test Fuzhou Co., Ltd, Fuzhou, 350003 Fujian China

**Keywords:** *Cerrena unicolor*, laccase, purification, Thermostability, Malachite green

## Abstract

Laccases are a class of multi-copper oxidases with important industrial values. A thermotolerant laccase produced by a basidiomycete fungal strain *Cerrena unicolor* CGMCC 5.1011 was studied. With glycerin and peptone as the carbon and nitrogen sources, respectively, a maximal laccase activity of 121.7 U/mL was attained after cultivation in the shaking flask for 15 days. Transcriptomics analysis revealed an expressed laccase gene family of 12 members in *C. unicolor* strain CGMCC 5.1011, and the gene and cDNA sequences were cloned. A glycosylated laccase was purified from the fermentation broth of *Cerrena unicolor* CGMCC 5.1011 and corresponded to Lac2 based on MALDI-TOF MS/MS identification. Lac2 was stable at pH 5.0 and above, and was resistant to organic solvents. Lac2 displayed remarkable thermostability, with half-life time of 1.67 h at 70 ºC. Consistently, Lac2 was able to completely decolorize malachite green (MG) at high temperatures, whereas Lac7 from *Cerrena* sp. HYB07 resulted in accumulation of colored MG transformation intermediates. Molecular dynamics simulation of Lac2 was conducted, and possible mechanisms underlying Lac2 thermostability were discussed. The robustness of *C. unicolor* CGMCC 5.1011 laccase would not only be useful for industrial applications, but also provide a template for future work to develop thermostable laccases.

## Key points

1. *C. unicolor* strain CGMCC 5.1011 produced 122 U/mL laccase.
2. The strain contained 12 expressed laccase isozymes.
3. A novel, thermostable laccase, Lac2, was purified and characterized.
4. Lac2 decolorized malachite green at 50 and 70 ºC whereas another laccase failed.

## Introduction

Laccases (EC 1.10.3.2) are copper-containing oxidases catalyzing oxidation of phenolic/non-phenolic lignin-related compounds and recalcitrant environmental pollutants (Baldrian 2006; Couto and Herrera 2006). Because laccases have low substrate specificity, utilize oxygen as final electron acceptor and produce water as only by-product, they find applications in paper pulping and bleaching, textile refining, dye decolorization, bioremediation, organic synthesis, juice and wine clarification, etc. (Ai et al. 2015; Yang et al. 2017a) Nonetheless, laccase applications are hampered by low production yields and reduced performance under industrial conditions such as high temperatures (Yang et al. 2017a).

Laccases are widespread in nature; they are found in microorganisms, plants and animals, and white-rot fungi are considered the most efficient laccase producers (Arora and Sharma 2010; Couto and Toca-Herrera 2007). Although the *Cerrena* genus is not as intensively studied as *Trametes*, *Cerrena* species have gained attention as laccase producers (Chen et al. 2012; Yang et al. 2017a). We have previously reported a *Cerrena* sp. HYB07 with high laccase yields; Lac7 is the laccase predominantly produced by HYB07 (Yang et al. 2014, 2015a, 2016b) and is active towards a wide range of substrates, including dyestuffs and antibiotics (Yang et al. 2015b, 2016a, 2016c, 2016d). In the present work, we continued our quest for laccase-secreting *Cerrena* and investigated a *C. unicolor* strain CGMCC 5.1011. Transcriptomics and cloning revealed a laccase gene family of 12 members in *C. unicolor* CGMCC 5.1011. Lac2 was purified from the fermentation broth and displayed extraordinary thermostability. Lac2 was able to decolorize the triphenylmethane dye malachite green (MG) despite incomplete decolorization by Lac7 from *Cerrena* sp. HYB07 due to heat inactivation. The research presented herein provided a novel laccase with thermotolerance and thermostability that would be desirable for industrial applications.

## Materials and methods

### Strain and media

*C. unicolor* strain CGMCC 5.1011 was purchased from China General Microbiological Culture Collection Center and maintained on potato dextrose agar (PDA) at 4 ºC. The fermentation medium for strain 5.1011 contained (g/L): KH_2_PO_4_ 6 g, MgSO_4_·7H_2_O 4.14 g, CaCl_2_ 0.3 g, NaCl 0.18 g, CuSO_4_·5H_2_O 0.0625 g, ZnSO_4_·7H_2_O 0.018 g, VB1 0.15 g and respective carbon and nitrogen sources. The following carbon sources were tested at 2.0% (w/v): Mannitol, glycerol, glucose, lactose, sucrose, cellulose, maltodextrin, corn dextrin, β-dextrin, soluble starch, corn starch, in combination with 1.5% (w/v) peptone. Next, the following nitrogen source were tested at the level of 1.5% (w/v): ammonium nitrate, ammonium tartrate, peptone, yeast extract, beef extract, ammonium sulphate, and soybean cake powder. The concentrations of the nutrient sources were also optimized. Fermentation of *Cerrena* sp. strain HYB07 was carried out as described (Yang et al. 2015a).

### Transcriptomics analysis of *C. unicolor* CGMCC 5.1011

Mycelia were collected from 6-d-old *C. unicolor* CGMCC 5.1011, and total RNA was extracted with a RNeasy Plant Mini Kit (Qiagen, Hilden, Germany). Transcriptomics analysis was performed by Novogene (Beijing, China). Briefly, after RNA quality check, 3 µg RNA was used as input material. Sequencing libraries were generated using NEBNext Ultr RNA Library Prep Kit for Illumina (NEB, Ipswich, MA, USA). After cluster generation, the library preparations were sequenced on an Illumina Hiseq platform and paired-end reads were generated; then data analysis, gene function was annotated based on the following databases: Nr, Nt, Pfam, KOG/COG, Swiss-Prot, KO, and GO.

### Cloning of laccase genes and cDNA

DNA was extracted with E.Z.N.A. HP Fungal DNA Kit (Omega, Norcross, GA, USA). TransScript One-Step Removal and cDNA Synthesis SuperMix (TransGen Biotech, Beijing, China) was used to synthesize the first strands of cDNA. PCR was carried out with 2 × EasyTaq PCR SuperMix (TransGen Biotech, Beijing, China). Thermal asymmetric interlaced PCR (TAIL-PCR) was used to clone the flanking sequences of incomplete laccase genes. Primers used are listed in Supplementary Table S1. PCR products were inserted into pMD18-T vector (Takara, Dalian, China), and the recombinant vectors were transformed into *E. coli* TOP10 competent cells (Life Technologies, Grand Island, NY, USA). Four clones of each PCR product were randomly selected and submitted to sequencing analysis.

### Bioinformatic analysis

Sequences were analyzed by using BLAST (Altschul 1990). Signal peptide was predicted with SignalP 3.0 (Bendtsen et al. 2004). Potential *N*-glycosylation sites (Asn-X-Ser/Thr) were identified with ScanProsite (Edouard et al. 2006). Alignments of laccase proteins were generated with Clustal Omega (Sievers et al. 2011). Phylogeny tree of selected fungal laccases were calculated in MEGA version 7.0 (Tamura 2011). Hydrophobic interaction and salt bridges were predicted by Protein Interactions Calculator (PIC) web server (Tina et al. 2007). Three-dimensional structures were visualized and analyzed by using PyMOL Molecular Graphics System (Version 1.80, Schrödinger, LLC) (Lam 2016).

### Enzyme activity assay

Laccase activity was assayed with ABTS (ε = 36,000 M^−1^ cm^−1^), guaiacol (ε = 26,600 M^−1^ cm^−1^) or catechol (ε = 1260 M^−1^ cm^−1^) as the substrate by following absorbance change at 420, 470 and 400 nm, respectively. One unit of enzyme activity was defined as the amount of enzyme needed to oxidize 1 μmol substrate in 1 min. All measurements were carried out in triplicate.

### Protein purification and characterization

The fermentation broth was harvested by centrifugation at 12,000*g* for 10 min and then filtered. The precipitate formed with 50% to 90% (NH_4_)_2_SO_4_ was collected by centrifugation (20,000*g*, 20 min), resuspended in buffer A (50 mM Tris–HCl buffer, pH 8.0) and dialyzed in buffer A. The dialyzed crude enzyme solution was applied at 5 mL/min to a HiTrap DEAE column pre-equilibrated with buffer A. Adsorbed proteins were eluted with 0.15 NaCl in buffer A. The purified protein was analyzed by SDS-PAGE for homogeneity and stained with Coomassie Brilliant Blue R-250. Deglycosylation was carried out with Peptide *N*-glycosidase F (Takara, Dalian, China) according to manufacturer’s instructions. Protein identification with MALDI-TOF MS/MS was performed by APT SHANGHAI Applied Protein Technology (Shanghai, China).

### Enzymatic characterization of the purified laccase

The effect of pH on laccase activity was determined between pH 2.5 to 6.5 at 40 ºC. pH stability was studied by incubating the enzyme at pH 2.5–9.0 at 30 ºC for 48 h. Residual laccase activity was quantified with ABTS as the substrate. Buffers used included citrate–phosphate buffer (pH 2.5–8.0), and glycine–NaOH buffer (pH 9.0).

For optimum temperature, laccase activity was measured at the optimum pH and temperatures from 20 to 70 ºC. Thermostability was analyzed by incubating the enzyme at different temperatures (40–70 ºC), and residual activity was assayed with ABTS at optimum pH and temperature. All experiments were performed in triplicate.

Effect of metal ions on activity of the purified enzyme was investigated. Metal ions Al^3+^, Ca^2+^, Ce^3+^, Cu^2+^, Fe^2+^, K^+^, Li^+^, Mg^2+^, Mn^2+^ and Zn^2+^ were in form of sulfate, Cd^2+^, Hg^2+^, Ni^2+^ and Co^2+^ in form of nitrate, and Pb^2+^ in form of subacetate. Individual inhibitor or metal ion was incorporated in the enzyme assay, and activity was determined with ABTS at optimal temperature and pH. Enzyme activity in absence of metal ions was regarded as 100%.

Organic solvents, namely methanol, ethanol, acetone, isopropanol, acetonitrile and dimethyl sulfoxide (DMSO), was added individually to the enzyme activity assay to the final concentration of 10% or 25%, and the laccase activity assay was carried out at the optimal temperature and pH with ABTS as the substrate. Enzyme activity in absence of organic solvents was regarded as 100%.

### MG decolorization

Decolorization of MG was carried out at 30, 50 and 70 °C with laccase from *C. unicolor* strain CGMCC 5.1011 and *Cerrena* sp. HYB07, respectively. The decolorization mixture contained 50 mM citrate–phosphate buffer (pH 6.0), 100 mg/L MG and 20 U/mL laccase. The mixture with heat-inactivated laccase was used as the negative control. After decolorization, the reaction mixtures were subjected to UV–visible analysis with a Hitachi U-2910 UV–Vis spectrophotometer (Chiyoda, Tokyo, Japan). Decolorization efficiency was monitored at 618 nm and calculated with the following formula:

$${\text{Decolorization efficiency }}\left( \%  \right)\, = \,\left( {{\text{A}}_{0} \, - \,{\text{A}}_{{\text{1}}} } \right)/{\text{A}}_{0} \, \times \,{\text{1}}00,$$

where A_0_ and A_1_ are the absorption of MG before and after laccase treatment, respectively.

### Molecular dynamics (MD) simulation

Homologous modeling was conducted with Phyre2 (Kelley et al. 2015), and MD simulation was carried out with BIOVIA Discovery Studio software (BIOVIA 2015). Solvation of the laccase protein was performed in CHARMm force field. Then standard dynamics cascade, including energy minimization for solvent, ions, protein and the whole system, heating, equilibration and MD production was done by the Discovery Studio software. The time for equilibration was 20 ps, and the time for MD production was 200 ps. The MD data was analyzed using Analyze Trajectory to obtain Root Mean Square Deviation (RMSD) and Root Mean Square Fluctuation (RMSF).

## Results

### Laccase production by *C. unicolor* CGMCC 5.1011

**Fig. 1:**
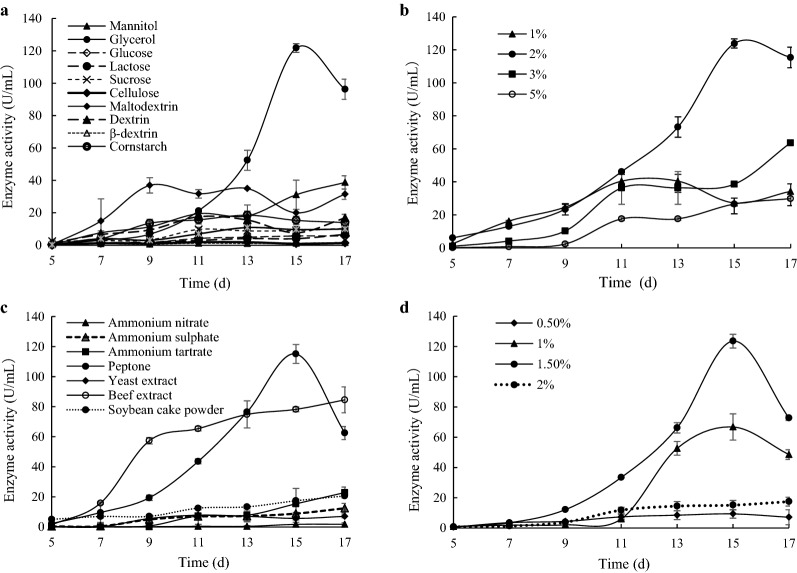
Laccase production by *C. unicolor* CGMCC 5.1011. **a** Effect of carbon sources on laccase production. **b** Effect of glycerol concentration on laccase production. **c** Effect of nitrogen sources on laccase production. **d** Effect of peptone concentration on laccase production

*C. unicolor* strain CGMCC 5.1011 was verified by 18 s rDNA sequencing. Among 11 carbon sources, glycerin was found to be the optimal carbon source, with the highest laccase activity of 121.7 U/mL at day 15 (Fig. 1a). Next, different concentrations of glycerin was used, and laccase production was followed (Fig. 1b). With 1.0% glycerin, the peak of 40.4 U/ml was reached at day 11. At higher glycerin concentrations of 3.0% and 5.0%, greatest laccase yields of 63.7 and 29.8 U/ml, respectively, were observed at day 17. Therefore, 2.0% was determined as the optimal glycerin concentration. Among the nitrogen sources tested, organic nitrogen sources were more effective than inorganic nitrogen sources. Peptone resulted in the highest activity, followed by beef extract, soybean cake powder, ammonium tartrate (Fig. 1c). Furthermore, 1.5% peptone resulted in the highest activity, followed by 1.0% (Fig. 1d).

### The laccase gene family of *C. unicolor* CGMCC 5.1011

**Fig. 2:**
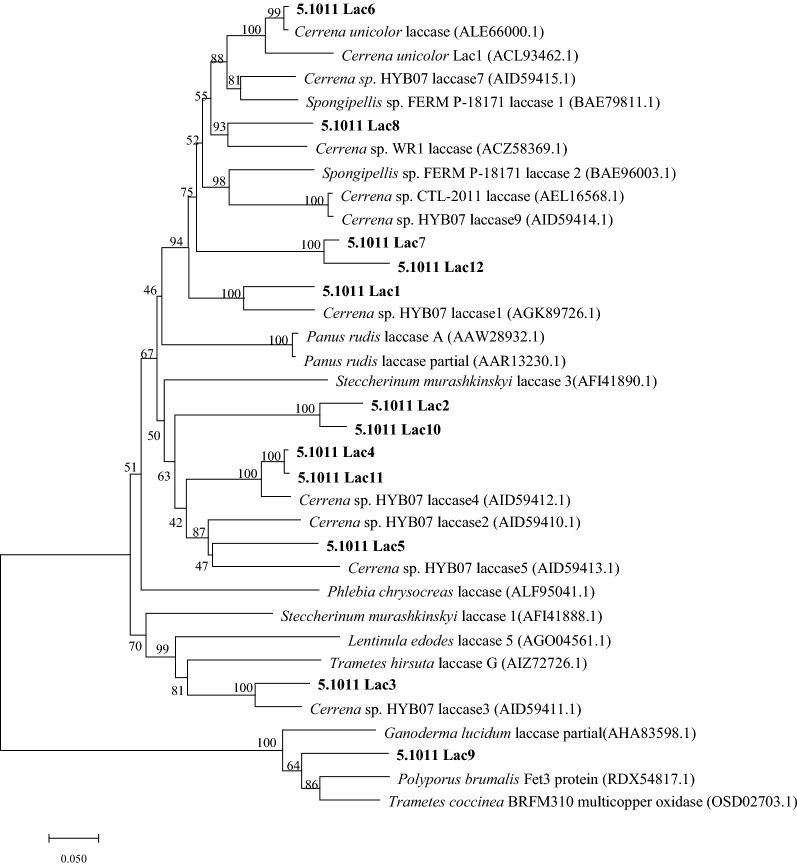
Phylogenetic relationship of *C. unicolor* CGMCC 5.1011 laccases with reported laccases. The scale bar indicates the estimated number of amino acid substitutions per site

Information on the transcriptome of *C. unicolor* CGMCC 5.1011 could be found in Additional file 1: Tables S2 and S3, and Fig. S1. A total of 12 laccase genes were identified and confirmed by the DNA and cDNA sequences (Table 1). The length of the nucleotide sequences for the twelve laccase genes ranged from 1652 (for *Lac12*) to 2406 bp (for *Lac9*). The deduced protein sequences of the 12 laccases were of length typical of fungal laccases (from 371 aa for Lac12 to 627 aa for Lac9), and all were predicted to contain a signal peptide. The intron numbers of the laccase isozymes varied between 6 and 12. A phylogenetic tree was constructed to show the evolutionary relationship of CGMCC 5.1011 laccases with reported laccases (Fig. 2).

**Table 1:** The *C. unicolor* CGMCC 5.1011 laccase gene family

Laccase	GenBank Accession No	DNA length (bp)	cDNA length (bp)	Intron number	Signal peptide (aa)	Mature protein (aa)	Glycosylation sites
Lac1	MT210509	2173	1554	11	20	497	3
Lac2	MT066188	2265	1599	12	20	512	13
Lac3	MT210510	1858	1509	6	20	482	8
Lac4	MT210511	2174	1548	11	19	496	4
Lac5	MT386937	2223	1599	11	27	505	9
Lac6	MT210513	2191	1551	11	21	495	3
Lac7	MT210514	2202	1551	11	21	495	11
Lac8	MT210515	2170	1551	11	21	495	5
Lac9	MT246201	2406	1884	9	17	610	13
Lac10	MT210516	2255	1596	12	20	511	12
Lac11	MT246202	1836	1323	9	20	419	4
Lac12	MT246203	1652	1116	8	14	357	9

### Laccase purification and characterization

**Fig. 3:**
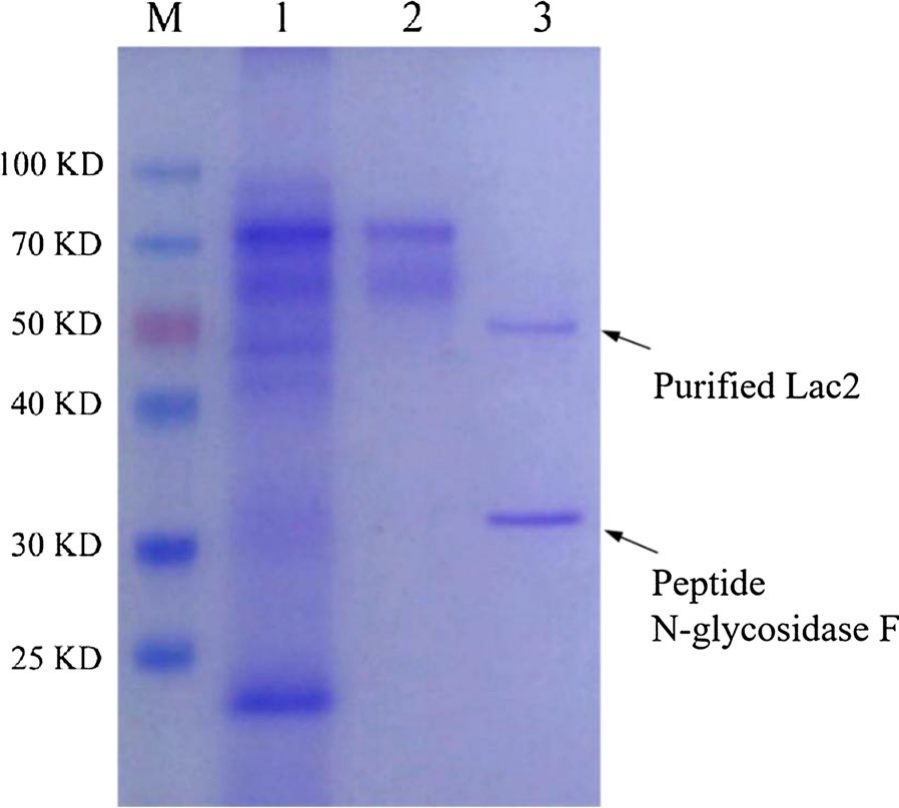
SDS-PAGE analysis of purified Lac2. Lac2 was purified from the fermentation broth of *C. unicolor* CGMCC 5.1011. Lane M, protein marker. Lane 1, fermentation broth of *C. unicolor* CGMCC 5.1011*.* Lane 2, purified Lac2. Lane 3, deglycosylated Lac2

A laccase was purified from CGMCC 5.1011 fermentation broth after (NH_4_)_2_SO_4_ precipitation and anion exchange chromatography. The protein appeared as a smear on SDS-PAGE between 58.4 and 74.0 kDa. After deglycosylation with peptide *N*-glycosidase F, a single band was shown around 50 kDa, indicating the laccase was heterogeneously glycosylated (Fig. 3). The glycosylation extent was estimated to be 16.8%-48.0%. The deglycosylated protein was subjected to MALDI-TOF MS/MS and was identified as Lac2 (Additional file 1: Fig. S2). The amino acid sequence of Lac2 was most similar to Lac4 from *Cerrena* sp. HYB07, sharing a 66% identity (Additional file 1: Table S4).

### Enzymatic properties of Lac2

**Fig. 4:**
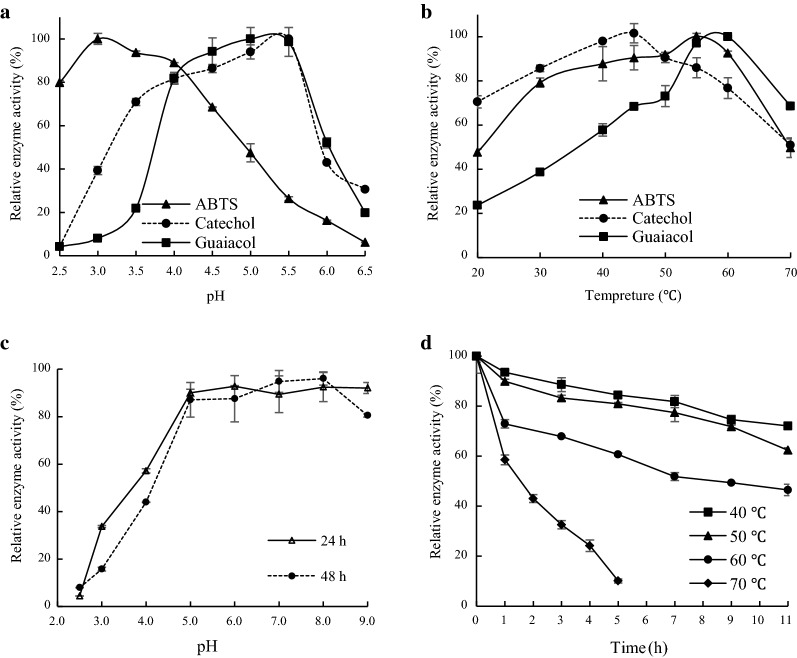
Effect of pH and temperature on Lac2 activity and stability. **a**, **b** Effect of pH and temperature on activity of Lac2. **c**, **d** Effect of pH and temperature on stability of Lac2

The pH optimum was 3.0 for ABTS and 5.5 for catechol and 5.0 for guaiacol (Fig. 4a). Optimal temperature was 55 ºC with ABTS, 45 ºC with catechol and 60 ºC with guaiacol. The enzyme displayed wide ranges of reacting temperatures, with > 50% of the maximal activity at 70 ºC against the three substrates (Fig. 4b).

At pH 5.0 or higher, > 80% activity remained after 48 h. In contrast, Lac2 was less stable at pH 2.5–4 (Fig. 4c). Lac2 was also stable at different temperatures. After incubation for 11 h at 40, 50 and 60 ºC, approximately 80, 70% and 50% of the original enzyme activity was retained, respectively (Fig. 4d). Heat inactivation rate *k* increased with temperatures, accompanied by reduced half-life time (*t*_1/2_) (Table 2).

**Table 2:** Thermal inactivation of *Cerrena* laccases

Strain	Laccase	Temperature (°C)	*t*_1/2_ (h)	*K* (h^−1^)	Reference
*Cerrena unicolor* CGMCC 5.1011	Lac2	40 50 60 70	22.02 16.85 7.79 1.67	0.032 0.041 0.089 0.414	This study
*Cerrena* sp. HYB07	Lac7	70	0.13	5.42	(Yang et al. 2017b)
*Cerrena unicolor* strain 137	Lacc I Lacc II	70	< 0.17 < 0.33	NR	(Michniewicz et al. 2006)
*Cerrena unicolor* C-139		70	0.25	NR	(Songulashvili et al. 2012)
*Cerrena unicolor* VKMF-3196	LacC1 LacC2	70	0.5 0.05	NR	(Zoya Alexandrovna et al. 2010)
*Cerrena* sp. WR1	Lcc3	50 60 70	2.0 0.67 0.13	NR	(Chen et al. 2012)
*Cerrena unicolor* BBP6	LacA	60 70	< 2.0 < 1.0	NR	(Ji et al. 2018)
*Cerrena* sp. RSD1	DLac	70	< 0.17	NR	(Wu et al. 2018)

ABTS was used as the substrate

**Fig. 5:**
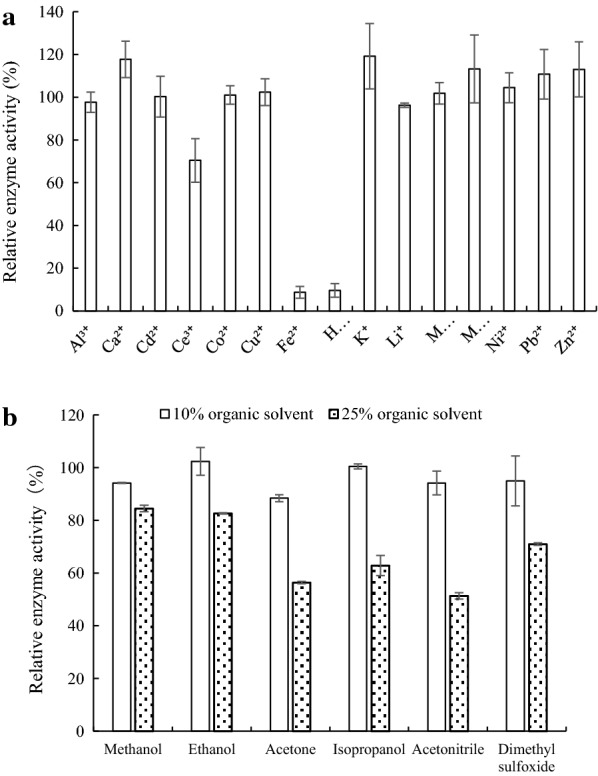
Effect of metal ions **a** and organic solvents **b** on Lac2 activity

Effect of metal ions and organic solvents on Lac2 activity was also studied. At 10 mM, Fe^2+^ and Hg^2+^ exerted the strongest inhibition on Lac2 activity, followed by Ce^3+^. On the other hand, Ca^2+^, K^+^, Mn^2+^, Pb^2+^ and Zn^2+^ were stimulatory, and the rest metal ions showed no significant effect (Fig. 5a).

When individual water-miscible organic solvent was added to the final concentration of 10%, Lac2 activity was similar to that in the absence of the organic solvents. Activity of Lac2 was compromised to different extents when the concentration was raised to 25%; > 80% activity was retained in methanol and ethanol, followed by DMSO, isopropanol and acetone, whereas acetonitrile resulted in lowest activity of approximately 50% (Fig. 5b).

### MG decolorization

To exemplify the operation stability offered by Lac2 thermostability, we compared Lac2 (from *C. unicolor* CGMCC 5.1011) and Lac7 (from *Cerrena* sp. HYB07) in MG decolorization, both in the absence of a redox mediator. We have previously shown MG is sequentially demethylated by laccase to colored intermediates desmethyl MG, didesmethyl MG, tridesmethyl MG and tetradesmethyl MG (Yang et al. 2015b).

**Fig. 6:**
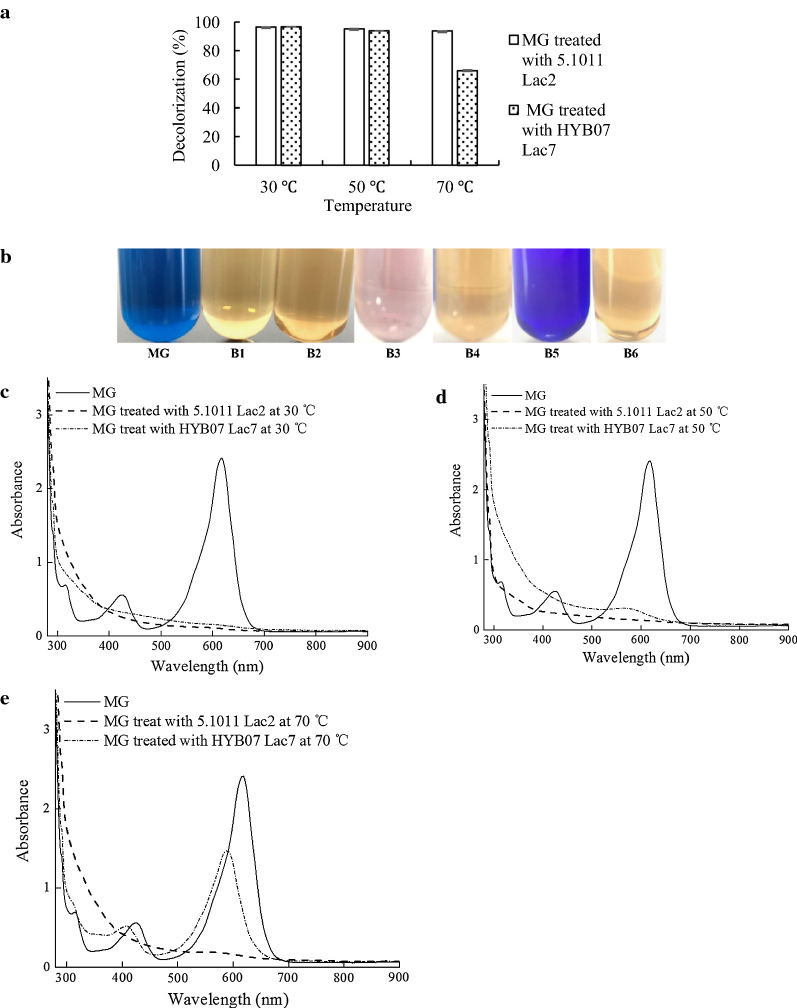
Laccase-mediated decolorization of MG. **a** Decolorization efficiency of MG by laccases at 30, 50 and 70 °C. **b** MG after laccases-mediated decolorization at different temperatures. B1, B3, B5: MG treated with HYB07 Lac7 at 30, 50 and 70 °C, respectively. B2, B4, B6: MG treated with CGMCC 5.1011 Lac2 at 30, 50 and 70 °C, respectively. **c**–**e** UV–vis spectra of MG before and after decolorization by laccases at 30 , 50 and 70 °C, respectively

At 30 ºC, two laccases performed similarly in MG decolorization (Fig. 6a–c). At 50 ºC, despite similar decolorization efficiencies calculated by absorbance decreases at 618 nm (Fig. 6a), HYB07 Lac7 resulted in a recalcitrant pink color, accompanied by a shift of the absorbance peak from 618 to 560 nm (Fig. 6b–c). This was caused by accumulation of a stable, colored intermediate tetradesmethyl MG due to instability of the HYB07 Lac7 and thus perturbation of the MG demethylation pathway (Yang et al. 2017b). This phenomenon was not observed with *C. unicolor* CGMCC 5.1011 Lac2.

At 70 ºC, CGMCC 5.1011 Lac2 still accomplished MG decolorization without intermediate accumulation. HYB07 Lac7, on the contrary, was quickly inactivated and barely decolorized MG (Fig. 6b and e). In fact, the shift of the absorbance peak to 590 nm was characteristic of a MG transformation product didesmethyl MG (Cho et al. 2003).

## Discussion

In this study, *C. unicolor* CGMCC *5.1011* could produce high activity laccase and probably secreted the enzyme by secondary metabolism. This is thought to be an energy-saving response common in laccase-producing fungi (Piscitelli et al. 2011; Yang et al. 2016b). Glycerin was the best carbon source for strain CGMCC 5.1011, and it also allowed for significant accumulation of laccase by *Cerrena maxima*, *Fomes fomentarius* and *Pseudotrametes gibbosa*. Some fungal strains prefer readily utilizable carbon sources. For example, HYB07 laccase expression is nearly abolished in the presence of glycerin as the sole carbon source (Yang et al. 2015a).

Fungal laccases exist in gene families, which may be derived from duplication-divergence events of a small set of ancestral enzymes. Laccase gene families have been analyzed in *Pleurotus ostreatus*, *Lentinula edodes, Coprinopsis cinerea*, etc.; the family size varies from 5 to 17 (Yang et al. 2017a). The laccase family of *Cerrena* sp. HYB07 is composed of 13 laccase genes (Li 2017). Laccase genes of these two *Cerrena* species were similar in terms of size, intron number, and presence of signal peptide. However, the amino acid sequences of CGMCC 5.1011 laccases seemed to contain more putative glycosylation sites (Asn-X-Ser/Thr, where X stands for any amino acid except for proline) than HYB07 laccases. CGMCC 5.1011 laccases were predicted to have 3–13 glycosylation sites per protein, with Lac2 having 13, while HYB07 laccases were predicted to contain 0–6 glycosylation sites per protein.

The major laccase secreted by CGMCC 5.1011, Lac2, was purified, and the enzymatic properties of purified Lac2 were studied. The optimal pH and pH stability of Lac2 were similar to those of reported fungal laccases, including Lac7 from *Cerrena* sp. HYB07 (Yang et al. 2014). On the other hand, the optimal reacting temperature of Lac2 was higher than that of Lac7. Hg^2+^, Fe^2+^, and Ce^3+^ inhibit activity of both CGMCC 5.1011 Lac2 and HYB07 Lac7; however, Pb^2+^ and Li^+^ did not suppress Lac2 activity as they do to Lac7 (Xu et al. 2018; Yang et al. 2014). Lac2 was more tolerant of organic solvents compared with a thermo-active and thermostable laccase, Lac37 II, from *Trametes trogii*. In particular, approximately only 40% activity of Lac 37 II remains in the presence of 10% methanol (Yang et al. 2020).

Most remarkably, at 70 ºC, *t*_1/2_ of Lac2 was 1.67 h, which was longer than many reported laccases (Chen et al. 2012; Ji et al. 2018; Michniewicz et al. 2006; Songulashvili et al. 2012; Wu et al. 2018; Zoya Alexandrovna et al. 2010) and comparable to a thermostable laccase Lac37 II from *T. trogii* (Yang et al. 2020). A comparison of thermal inactivation of *Cerrena* laccases is provided in Table 2. The predominant laccase from *Cerrena* sp. HYB07, Lac7, was stable at 60 ºC and below, but was completely inactivated in 20 min at 70 ºC (Yang et al. 2014). The differences in thermostability of Lac2 from *C. unicolor* CGMCC 5.1011 and Lac7 from *Cerrena* sp. HYB07 could also be seen in their fluorescence spectra collected after incubating the respective proteins for 1 h at 30, 40, 50, 60, and 70 ºC (Additional file 1: Fig. S3). With increased incubation temperatures, fluorescence spectra of Lac7 demonstrated significant decreases and red shift in fluorescence intensity at the emission peak, suggesting the protein was prone to lose its natural conformation at higher temperatures. In contrast, the fluorescent emission peaks of Lac2 remained relatively constant at different temperatures, consistent with its higher thermostability.

There are several putative molecular mechanisms underlying protein thermotolerance or thermostability (Hilden et al. 2009). An amino acid alignment of *C. unicolor* CGMCC 5.1011 Lac2 and *Cerrena* sp. HYB07 Lac7 showing the four conserved fungal laccase copper-binding signature domains (L1–L4) and six substrate-binding loops (B1–B2, B4–B5, B7–B8, C1–C2, C4–C5, and C7–C8) is provided in Additional file 1: Fig. S5. We speculated that the thermostability of Lac2 can at least be partially attributed to its high glycosylation content. Glycosylation is suggested to have a general nonspecific effect on enzyme stabilization (Manuel et al. 2015; Shental-Bechor and Levy 2008; Vite-Vallejo et al. 2009). It has been reported that the high carbohydrate level (49%) protected a *Botrytis cinerea* laccase from high-temperature denaturation (Slomczynski et al. 1995). Increased thermostability of a recombinant laccase (rLac) produced by *Pichia pastoris* compared to the native laccase (nLac) is also attributed to higher glycosylation, and the glycan moieties played a crucial role in the laccase activity (Garg et al. 2012). In this study, among the 13 potential glycosylation sites identified in CGMCC 5.1011 Lac2, 12 were found on the protein surface except for N71, which was near the protein surface and at the back of the active center (Additional file 1: Fig. S4). In contrast, only one putative glycosylation site was found on the surface of Lac7 from *Cerrena* sp. HYB07, which has a 7.2% carbohydrate content (Yang et al. 2014), and Lac7 was inferior to Lac2 in CGMCC 5.1011 with regards to stability and performance at higher temperatures.

Hydrophobic interactions play a governing role in stabilizing the protein 3D structure (Christensen and Kepp 2012). Pace et al. argued that hydrophobic interactions made the most contribution to protein stability through his study of 22 proteins (Pace et al. 2011). Hydrophobic interaction is also presumably a basis for the disparate thermostability between two adenylate kinases sharing 78% sequence identity (Criswell et al. 2003). More hydrophobic interactions (within 5 Å) were found in Lac2 compared with Lac7 (438 vs. 396), which might lead to higher thermostability of Lac2.

**Fig. 7:**
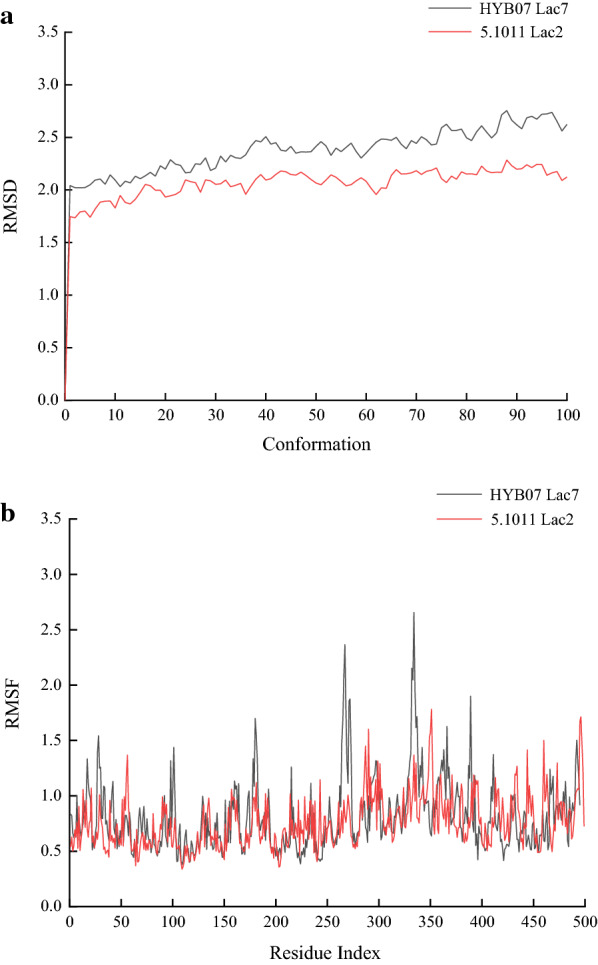
Molecular Simulation of Lac2 from *C. unicolor* CGMCC 5.1011 and Lac7 from *Cerrena* sp. HYB07. **a** RMSD values of the laccases. **b** RMSF values of the laccases

The MD simulation of the two laccases also shined light on the molecular determinants of Lac2 thermostability. The overall RMSD value of Lac2 were lower than that of Lac7, indicating the whole flexibility of Lac2 was smaller than Lac7 (Fig. 7a). Comparison of the RMSF values of the two laccases revealed regions with significantly higher RMSF values in Lac7 than in Lac2 (Fig. 7b), especially the 180^th^ residue near the substrate-binding loop B1–B2, 267th residue in B7–B8 and 334th residue in C1–C2, corroborating the lower flexibility and higher stability of Lac2. Residues 98–102, upstream to the fungal laccase signature domain L2, were associated with lower RMSF values in Lac2, meaning they might help maintain the rigidity of the L2. Regions 262–273 and 331–342 corresponded to substrate-binding loops B7–B8 and C1–C2, respectively, they showed higher RMSF values in Lac7 than in Lac2. A unique salt bridge in Lac2, His326-Asp340, flanking the substrate-binding loop C1–C2, might contribute to lower the flexibility around loop C1–C2 in Lac2, whereas the majority of other salt bridges in the two proteins were conserved (Additional file 1: Table S5). Meanwhile, region 366–389 in Lac7 overlapped with the substrate-binding loop C4–C5 and neighbored the conserved laccase signature domain L3, with residues 384–389 in loop C4–C5 (Additional file 1: Fig. S5). The RMSF values, 1.9009 for Leu389 in Lac7 and 0.9833 for Ile393 in Lac2 (Fig. 7b), indicated that the region might also offer rigidity to protect the Lac2 active site against heat. The above findings suggested higher rigidity in various substrate-binding loops as well as conserved laccase signature domains in Lac2 allows it to retain more activity under high temperatures.

In summary, a white-rot fungal strain *C. unicolor* CGMCC 5.1011 achieved a maximum activity of 121.7 U/mL after fermentation for 15 d. Strain CGMCC 5.1011 contained 12 laccase isozymes, and a major laccase, Lac2, was purified from the fermentation broth. Lac2 was reactive over a wide range of temperatures, was pH- and temperature-stable and tolerant of organic solvents. Lac2 decolorized MG whereas a less thermostable laccase failed, corroborating the thermostability and operational stability of this novel laccase. High-level glycosylation and structural rigidity might account for the high stability of Lac2.

## Supplementary information

**Additional file 1.** Additional figures and tables.

## Data Availability

All the data were presented in the main paper.
