# Supplementary material for: A highly thermotolerant laccase produced by Cerrena unicolor strain CGMCC 5.1011 for complete and stable malachite green decolorization
Source: AMB Express. 2020 Oct 2;10:178. doi: 10.1186/s13568-020-01118-z (PMC7532254; doi:10.1186/s13568-020-01118-z)
Supplement: Supplementary file 1 — Additional file 1. Additional figures and tables. [file 13568_2020_1118_MOESM1_ESM.docx]

**Additional data for**

**A highly thermotolerant laccase produced by *Cerrena unicolor* strain CGMCC 5.1011 for complete and stable malachite green decolorization**

Yanhua Yao^1^, Guimei Zhou^1^, Yonghui Lin^2^, Xinqi Xu^1*^, Jie Yang^1*^

^1^*Fujian Key Laboratory of Marine Enzyme Engineering, College of Biological Sciences and Technology, Fuzhou University, Fuzhou, Fujian 350116, China*

^2^*GRG Metrology & Test Fuzhou Co., Ltd., Fuzhou, Fujian 350003, China*

Table S1 Primers used in this study.

| Primer | Oligonucleotide sequence | Note |
| --- | --- | --- |
| Lac1-F | 5’-CATGAAGAGACACACCTCCATAG-3’ | Amplification of Lac1 cDNA and DNA |
| Lac1-R | 5’-CTACAAGTCCTTGCTATCTAGTGC-3’ |  |
| Lac2-F | 5’-ATGGGCGTGGGTCTACTCTC-3’ | Amplification of Lac2 cDNA and DNA |
| Lac2-R | 5’-TCATGATGCACTGGTCTGAGC-3’ |  |
| Lac3-F | 5’-TGTTTAACTTTTATTCTTTGCTAAGTTTCG-3’ | Amplification of Lac3 cDNA and DNA |
| Lac3-R | 5’-TTACTGGTCAGCGGAGTCAAG-3’ |  |
| Lac4-F | 5’-ATGCTTTGGCGTACCACTCTCAC-3’ | Amplification of Lac4 cDNA and DNA |
| Lac4-R | 5’-TCATGTATCACCTGGGCTCAAG-3’ |  |
| Lac5-F | 5’-CGATGGTATATAAGACGATGATGGGATTGAACTC-3’ | Amplification of Lac5 cDNA and DNA |
| Lac5-R | 5’-GTTTAAATAGCAGTTCCTTTCTTAGGCTTGAC-3’ |  |
| Lac6-F | 5’-AATGGTCTTCAGCGCTGCATTC-3’ | Amplification of Lac6 cDNA and DNA |
| Lac6-R | 5’-CGTTTACTTGTCACCGTCAGC-3’ |  |
| Lac7-F | 5’-ATGTACTACTGGTCTCCACTAGG-3’ | Amplification of Lac7 cDNA and DNA |
| Lac7-R | 5’-GATGTCCTTCAATGCAGCCTTC-3’ |  |
| Lac8-F | 5’-GCACAATGTTGTTCTGCTCTGC-3’ | Amplification of Lac8 cDNA and DNA |
| Lac8-R | 5’-GCTACTTGTCACCGTCATCG-3’ |  |
| Lac9-F | 5’-ATGTGGTCCTCCTCCCTCGC-3’ | Amplification of Lac9 cDNA and DNA |
| Lac9-R | 5’-TTACGCCTTGGGCTTCAGGAGA-3’ |  |
| Lac10-F | 5’-ATGGGCGTGGGTCTACTCTCA-3’ | Amplification of Lac10 cDNA and DNA |
| Lac10-R | 5’-CTACACACCGGTCTGGGTTGG-3’ |  |
| Lac11-F | 5’-GATGTCTCTTCTTCGTAGCTTGAC-3’ | Amplification of Lac11 cDNA and DNA |
| Lac11-R | 5’-ATGCTCAAGGCCACGTCCATTC-3’ |  |
| Lac12-F | 5’-GATGGGCCTGCTTTTTCATTTATCCACTC-3’ | Amplification of Lac12 cDNA and DNA |
| Lac12-R | 5’-CCGATGTACTACTGGTCTCCACTAG-3’ |  |
| TAIL-AD1 | 5’-TGWGNAGWANCASAGA-3’ | Degenerate primers for TAIL-PCR |
| TAIL-AD2 | 5’-AGWGNAGWANCAWAGG-3’ |  |
| TAIL-AD3 | 5’-STTGNTASTNCTNTGC-3’ |  |
| TAIL-AD4 | 5’-NTCGASTWTSGWGTT-3’ |  |
| TAIL-AD5 | 5’-NGTCGASWGANAWGAA-3’ |  |
| TAIL-AD6 | 5’-WGTGNAGWANCANAGA-3’ |  |
| TAIL-AD7 | 5’-WTCTGNCTWANTANCT-3’ |  |
| Lac3 SP1 | 5’-cattgacaaccacaccatgaccatcatcg-3’ | Amplification of 3’-flanking sequence of Lac3 |
| Lac3 SP2 | 5’-caccgctaaccagactgtcgacaactactg-3’ |  |
| Lac3 SP3 | 5’-ccaccagcactgctgctctcgaccagactgc-3’ |  |
| Lac5 SP1 | 5’-accaaacggaatcggtatcgtttgttatg-3’ | Amplification of 5’-flanking sequence of Lac5 |
| Lac5 SP2 | 5’-cgacaacggtctgggcgagaacatggtac-3’ |  |
| Lac5 SP3 | 5’-cacagtactgcgtggataaatgactatgg-3’ |  |

Table S2 Sequencing output data quality assessment.

| Sample | Raw Reads | Clean reads | Clean bases | Error (%) | Q20 (%) | Q30 (%) | GC (%) |
| --- | --- | --- | --- | --- | --- | --- | --- |
| 5.1011 | 59856856 | 58154292 | 8.72 G | 0.02 | 97.53 | 93.23 | 51.51 |

Table S3 Statistics on success rate of gene annotation in seven databases.

| Database | Number of Genes | Percentage (%) |
| --- | --- | --- |
| NR | 23628 | 79.71 |
| NT | 4166 | 14.05 |
| KO | 6818 | 23 |
| SwissProt | 15353 | 51.79 |
| PFAM | 19074 | 64.34 |
| GO | 19097 | 64.42 |
| KOG | 9833 | 33.17 |
| All databases | 2296 | 7.74 |
| At least one database | 24905 | 84.01 |

Table S4 Amino acid sequence identities of *Cerrena* laccases.

|  |  | *C. unicolor* 5.1011 | | | | | | | | | | *Cerrena* sp. HYB07 | | | | | | | | | | | | |
| --- | --- | --- | --- | --- | --- | --- | --- | --- | --- | --- | --- | --- | --- | --- | --- | --- | --- | --- | --- | --- | --- | --- | --- | --- |
|  |  | Lac2 | Lac4 | Lac5 | Lac6 | Lac7 | Lac8 | Lac9 | Lac10 | Lac11 | Lac12 | lac1 | lac2 | lac3 | lac4 | lac5 | lac6 | lac7 | lac8 | lac9 | lac10 | lac11 | lac12 | lac13 |
| *C. unicolor* 5.1011 | Lac1 | 59.2 | 69.8 | 65.9 | 73.8 | 69.3 | 73.2 | 23.0 | 60.0 | 60.7 | 46.6 | 85.1 | 68.8 | 66.0 | 69.8 | 64.5 | 55.4 | 74.1 | 69.4 | 63.0 | 69.0 | 60.7 | 46.2 | 68.0 |
|  | Lac2 |  | 66.2 | 66.5 | 60.9 | 59.4 | 58.4 | 22.9 | 90.2 | 57.0 | 37.2 | 59.2 | 65.7 | 55.7 | 65.5 | 62.9 | 56.2 | **61.4** | 58.4 | 62.7 | 62.7 | 79.5 | 59.0 | 56.6 |
|  | Lac3 |  | 62.9 | 57.8 | 66.5 | 60.9 | 61.7 | 25.1 | 56.6 | 57.3 | 41.8 | 63.5 | 60.7 | 86.7 | 63.0 | 57.0 | 53.0 | 65.9 | 63.7 | 57.2 | 56.8 | 57.6 | 41.8 | 59.2 |
|  | Lac4 |  |  | 72.2 | 72.3 | 67.1 | 71.8 | 21.2 | 67.6 | 84.5 | 43.5 | 68.1 | 74.2 | 63.5 | 93.0 | 67.5 | 57.8 | 72.3 | 67.2 | 69.9 | 68.4 | 69.7 | 68.1 | 65.8 |
|  | Lac5 |  |  |  | 66.7 | 63.4 | 67.7 | 23.3 | 69.2 | 62.5 | 39.5 | 64.9 | 77.6 | 60.0 | 72.8 | 73.7 | 58.9 | 67.9 | 63.6 | 78.4 | 73.8 | 68.1 | 64.5 | 60.3 |
|  | Lac6 |  |  |  |  | 73.3 | 77.3 | 23.4 | 63.0 | 63.8 | 49.8 | 73.9 | 70.3 | 69.8 | 72.3 | 67.2 | 57.8 | 86.1 | 76.2 | 66.6 | 65.3 | 64.4 | 79.8 | 70.1 |
|  | Lac7 |  |  |  |  |  | 73.8 | 25.0 | 60.6 | 58.2 | 65.3 | 69.5 | 65.0 | 62.2 | 68.1 | 62.5 | 54.7 | 75.0 | 70.2 | 64.5 | 60.6 | 58.2 | 74.6 | 80.2 |
|  | Lac8 |  |  |  |  |  |  | 22.6 | 62.5 | 62.9 | 48.3 | 72.2 | 69.8 | 63.5 | 71.6 | 65.3 | 56.9 | 78.3 | 71.7 | 66.6 | 63.8 | 65.0 | 83.0 | 69.8 |
|  | Lac9 |  |  |  |  |  |  |  | 22.9 | 21.3 | 16.7 | 23.0 | 23.2 | 24.8 | 23.2 | 23.0 | 24.6 | 23.1 | 24.1 | 24.0 | 23.0 | 24.0 | 23.8 | 22.7 |
|  | Lac10 |  |  |  |  |  |  |  |  | 59.6 | 38.3 | 59.9 | 67.2 | 56.8 | 67.1 | 65.7 | 57.6 | 63.6 | 58.1 | 64.1 | 63.6 | 82.5 | 59.9 | 58.2 |
|  | Lac11 |  |  |  |  |  |  |  |  |  | 50.5 | 59.6 | 65.8 | 54.8 | 80.6 | 59.1 | 50.3 | 62.9 | 58.2 | 60.8 | 61.7 | 60.3 | 58.4 | 57.5 |
|  | Lac12 |  |  |  |  |  |  |  |  |  |  | 46.8 | 41.1 | 39.7 | 44.0 | 39.2 | 35.6 | 50.4 | 45.6 | 41.8 | 40.7 | 40.2 | 50.0 | 53.0 |

Table S5 Predicted salt bridges in the laccases.

| Salt bridge No. | 5.1011 Lac2 | HYB07 Lac7 |
| --- | --- | --- |
| 1 | 18ASP-153HIS | 18ASP-153HIS |
| 2 | 22ARG-118ASP | 22ARG-118ASP |
| 3 | 66HIS-426ASP | 66HIS-422ASP |
| 4 | 121ARG-150ASP | 121ARG-150ASP |
| 5 | 131ASP-196ARG | 131ASP-197ARG |
| 6 | 138ASP-194ARG | 138ASP-195ARG |
| 7 | 140ASP-198ARG | 140ASP-199ARG |
| 8 | 196ARG-221GLU | 197ARG-222GLU |
| 9 | 198ARG-221GLU | 199ARG-222GLU |
| 10 | 213ASP-259ARG | 214ASP-260ARG |
| 11 | 223ASP-242ARG | 224ASP-243ARG |
| 12 | 223ASP-425ARG | 224ASP-421ARG |
| 13 | 242ARG-426ASP | 243ARG-422ASP |
| 14 | 302GLU-425ARG | 302GLU-421ARG |
| 15 | 384GLU-440ARG | 381GLU-436ARG |
| 16 | 404HIS-469GLU | 400HIS-465GLU |
| 17 | 91ASP-490LYS | 175ARG-182ASP |
| 18 | 280ARG-286ASP | 175ARG-288GLU |
| 19 | 326HIS-340ASP |  |


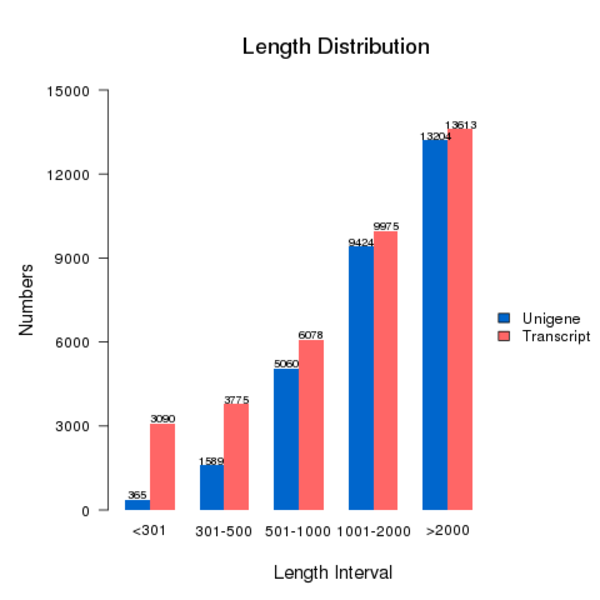


Fig. S1 Distribution of assembly length of the transcriptome of *C. unicolor* 5.1011.


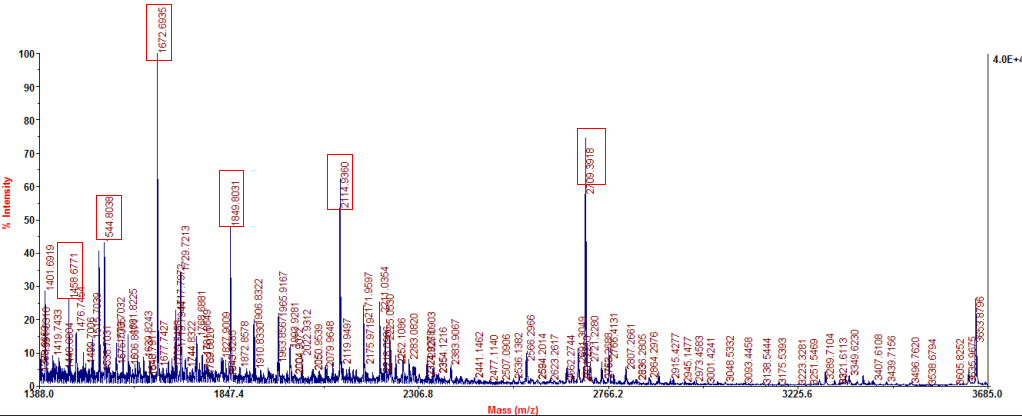


Fig. S2 The result of the MALDI-TOF MS/MS analysis of the purified Lac2 protein.

Lac2 was identified to be the protein product encoded by the *Lac2* gene.

|  |  |
| --- | --- |

Fig. S3 Fluorescence spectra of Lac2 from *C. unicolor* 5.1011 (left) and Lac7 from *Cerrena* sp. HYB07 (right).

The laccases were incubated at 30-70 ºC for 1 h; and then the fluorescence spectra were collected with an F-7000 fluorescence spectrophotometer (Hitachi, Japan) after excitation at 280 nm.


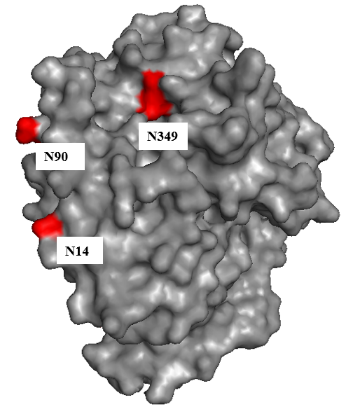

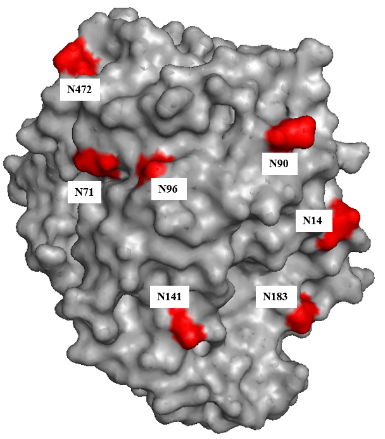

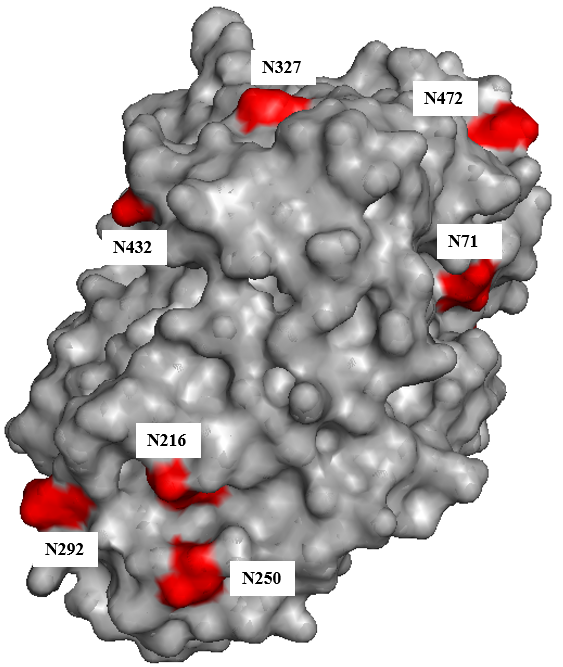


**90^o^**

**180^o^**

Fig. S4 3D structure display of putative Lac2 glycosylation sites


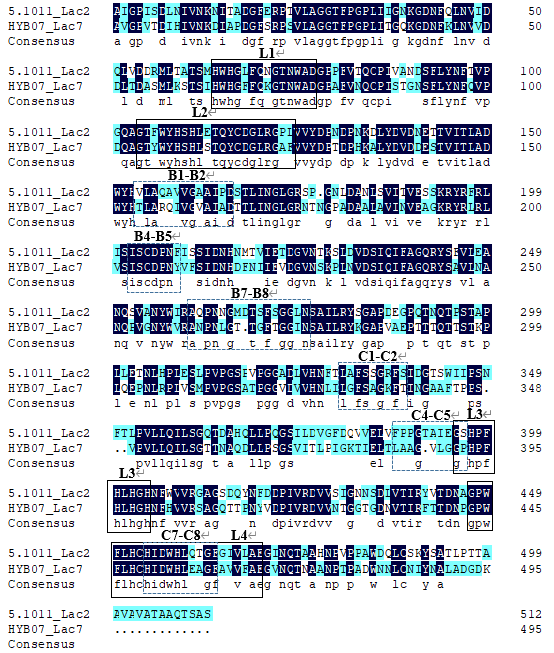


Fig. S5 Amino acid alignment of Lac2 of *C. unicolor* 5.1011 and Lac7 of *Cerrena* sp. HYB07.

The highly conserved laccase signature domains (L1-L4) are boxed in solid black lines. The substrate-binding loops are boxed with dotted lines.
